# Development and Characterization of 18 Novel EST-SSRs from the Western Flower Thrips, *Frankliniella occidentalis* (Pergande)

**DOI:** 10.3390/ijms13032863

**Published:** 2012-03-05

**Authors:** Xian-Ming Yang, Jing-Tao Sun, Xiao-Feng Xue, Wen-Chao Zhu, Xiao-Yue Hong

**Affiliations:** Department of Entomology, Nanjing Agricultural University, Nanjing 210095, Jiangsu, China; E-Mails: zqbxming@163.com (X.-M.Y.); 2009202023@njau.edu.cn (J.-T.S.); xfxue@njau.edu.cn (X.-F.X.); zzduke@126.com (W.-C.Z.)

**Keywords:** *Frankliniella occidentalis*, EST-SSRs, expressed sequence tags, microsatellite, population genetics

## Abstract

The western flower thrips, *Frankliniella occidentalis* (Pergande), is an invasive species and the most economically important pest within the insect order Thysanoptera. For a better understanding of the genetic makeup and migration patterns of *F. occidentalis* throughout the world, we characterized 18 novel polymorphic EST-derived microsatellites. The mutational mechanism of these EST-SSRs was also investigated to facilitate the selection of appropriate combinations of markers for population genetic studies. Genetic diversity of these novel markers was assessed in 96 individuals from three populations in China (Harbin, Dali, and Guiyang). The results showed that all these 18 loci were highly polymorphic; the number of alleles ranged from 2 to 15, with an average of 5.50 alleles per locus. The observed (*H*_O_) and expected (*H*_E_) heterozygosities ranged from 0.072 to 0.707 and 0.089 to 0.851, respectively. Furthermore, only two locus/population combinations (WFT144 in Dali and WFT50 in Guiyang) significantly deviated from Hardy–Weinberg equilibrium (HWE). Pairwise *F*_ST_ analysis showed a low but significant differentiation (0.026 < *F*_ST_ < 0.032) among all three pairwise population comparisons. Sequence analysis of alleles per locus revealed a complex mutational pattern of these EST-SSRs. Thus, these EST-SSRs are useful markers but greater attention should be paid to the mutational characteristics of these microsatellites when they are used in population genetic studies.

## 1. Introduction

The western flower thrips, *Frankliniella occidentalis* (Pergande), is the most economically important pest within the insect order Thysanoptera, which includes more than 5500 described species [1]. *F. occidentalis* causes enormous damage by directly feeding on greenhouse vegetable and ornamental crops and by transmitting plant-pathogenic tospoviruses [2]. *F. occidentalis* is endemic to North America in an area west of the Rocky Mountains from Mexico to Alaska [3]. Since the late 1970s, *F. occidentalis* has rapidly invaded most countries throughout the world where it not only causes severe economic losses but also threatens endemic invertebrates and associated ecosystems [4]. In order to control *F. occidentalis*, it is first necessary to know its genetic diversity, population structure and invasion history. Genetic tools, such as microsatellites markers, can reveal the origin of newly established populations, their genetic makeup and their routes of migration [5,6].

Microsatellites, or simple sequence repeats (SSRs), consist of tandemly repeated motifs that are 1–6 bp in length, and they are widely distributed throughout the eukaryotic genomes [7]. Conventionally, two models of mutations have been considered for microsatellites, the stepwise mutational model (SMM) and the infinite allele model (IAM). The SMM states that all mutational events involve a change in a single repeat only. The IAM assumes that every mutation results in the creation of a new allele [8]. The mutational mechanism of microsatellites is still under debate though it appears most likely to be slippage events during DNA replication [9]. Several other mechanisms may also be responsible for the generation of new alleles, e.g., insertions/deletions (indels) in the flanking region [10]. Matsuoka showed that the IAM model was appropriate for maize microsatellites mutated in the flanking regions [10]. Knowledge of the mutational pattern of one specific SSR could facilitate the selection of appropriate mutation model and combinations of markers in the population genetic studies. Currently, due to their codominant inheritance, highly polymorphic, easy detection by polymerase chain reaction (PCR) and broad distribution in the genome, microsatellites/SSRs are widely used for population genetic studies [11]. Ascunce *et al*. have used a large number of SSRs to investigate the global invasion route of the fire ant *Solenopsis invicta* [6]. However, population genetic studies of *F. occidentalis* have been hampered by a lack of polymorphic molecular markers. Presently, only 6 polymorphic microsatellites of *F. occidentalis* are known [12]. Recently, an enormous number of ESTs (expressed sequence tags) of *F. occidentalis* have become available in the public sequence database [13], and can be exploited to identify markers inexpensively. Hence, we isolated and characterized 18 novel EST-SSRs for *F. occidentalis*. These EST-SSRs will allow researchers to investigate the genetic diversity and population genetic structure of *F. occidentalis* in its native and invasive range and trace its global invasion history.

## 2. Results and Discussion

### 2.1. Characteristics of *F. occidentalis* EST-SSRs

We obtained 309 sequences containing SSRs by MIcroSAtellite (MISA) [14] analysis. Among these sequences, five contained two different SSRs and three of these were compound microsatellites (Table 1). The EST-SSR frequency (1SSR/24.1 kb) of *F. occidentalis* was smaller than that of brown planthopper (1SSR/13.0 kb; [15]), pea aphid (1SSR/3.0 kb; [16]) and several other insects (~1SSR/1 kb in fly, silkworm and mosquito; [17]) and was comparable to some crops (1SSR/23.80 kb in soybean and 1SSR/28.32 kb in maize; [18]). The most abundant repeat motif class was dinucleotide repeats (DNRs, 265/314). The AC/GT (41.5%) motif was the most common among DNRs, followed by AG/CT (31.7%), AT/AT (22.3%) and CG/CG (4.5%). The classification of repeats into classes was carried out according to the method of Jurka and Pethiyagoda [19]. For example, (AC)*n*, (CA)*n*, (TG)*n* and (GT)*n* were considered as the same class considering complementary sequences and/or different reading frames. Other repeat motifs, including trinucleotide, tetranucleotide, pentanucleotide, were also observed, albeit infrequently (49/314) (Table 1).

Of the primer pairs designed from 122 sequences suitable for primer design, 72 amplified the expected products, 50 yielded larger or no products. Finally, 18 primer pairs revealed polymorphism (Table 2), the remaining (54) were either monomorphic or amplified poorly. For these 18 selected ESTs, homology searches with the BLASTX against the NCBI nr database found three ESTs with significant hits to insect genes at an E-value cutoff level of 1e-5 (Table 2). No hit was found for any of the other 15 ESTs. Sequence length variation in coding sequences is rare. Examination of the three sequences with significant blast hits suggested that SSR sequences may be located on either 5′- or 3′-UTR (untranslated regions). The 15 remaining ESTs with unknown function may also originate from non-coding regions. These selected ESTs seem unlikely to come from non-insect sources because of the high amplification rates (approximately 99.5%) of these EST-SSRs across the 96 *F. occidentalis* samples. When analyzed all the published *F. occidentalis* ESTs, 17 of the 18 selected ESTs were singletons, the remaining one (GT306150) was part of a larger contig which contained only two ESTs. The low copy number of these ESTs suggested that they seem unlikely to be repetitive sequences in the nuclear genome. When considering all three populations (Table 3), 99 alleles were identified from 18 markers, the number of alleles (*N*a) ranged from 2 to 15, with an average of 5.50 alleles per locus. The observed (*H*_O_) and expected (*H*_E_) heterozygosities ranged from 0.072 to 0.707 and 0.089 to 0.851, respectively. The PIC values ranged from 0.088 to 0.860, with an average of 0.476 (Table 2).

After sequential Bonferroni correction for multiple tests, only WFT144 in Dali and WFT50 in Guiyang significantly deviated from Hardy-Weinberg equilibrium (HWE), possibly due to the presence of null alleles, which was further confirmed by the MICRO-CHECKER [20] analysis (Table 4). In addition, no band-stuttering, large allele dropouts or significant genotypic linkage disequilibrium was detected. Genetic diversity analysis indicated that Dali displayed the highest number of alleles (*N*a = 4.944) and expected heterozygosity (*H*_E_ = 0.522) and Harbin the lowest (*N*a = 4.389; *H*_E_ = 0.468) (Table 4).

### 2.2. Mutations of EST-SSRs

**Figure 1:**
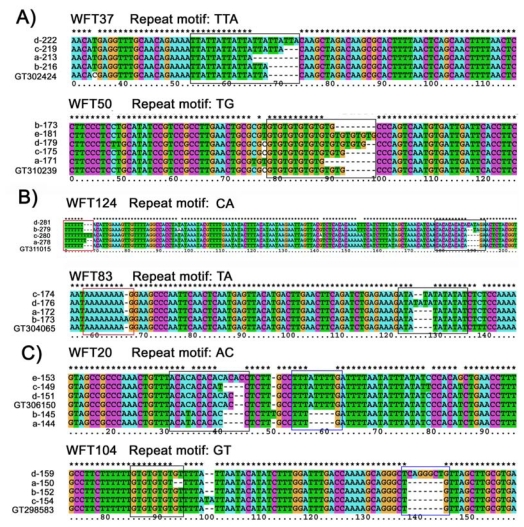
Mutational patterns of EST-SSRs in *Frankliniella occidentalis*. (**A**) microsatellites mutated in the repeat motif; (**B**) microsatellites which have one SSR in the flanking region; (**C**) microsatellites which have indels in the flanking region. Each base is indicated by a different color. The repeat motifs are shown in black box. The red box indicates the repeat motifs in the flanking region and the blue one indicates the indels in the flanking region.

Ninety-five different alleles, whose allele frequency was approximately 98%, were successfully sequenced. All the sequences obtained corresponded exactly to the expected EST sequences. The other 4 rare alleles with a low frequency were not sequenced. Sequence analysis of these alleles revealed that three types of mutational events are responsible for the generation of new alleles (Six loci exhibiting all three mutation patterns are listed in Figure 1, the other 12 are shown in Figures S1 and S2). First, size variation of sequenced alleles was explained by the differences in the numbers of repeat motifs for 7 microsatellites (Figure 1A, Figure S1). Second, at the WFT51, WFT83, WFT108 and WFT124 loci, two different repeat motifs, including one in the flanking region, were found, both contributing to the allele-size variation (Figure 1B, Figure S2A). Third, indels in the flanking region were observed in 7 loci (WFT20, WFT66, WFT87, WFT104, WFT139, WFT141 and WFT144; (Figure 1C, Figure S2B)), but the frequencies of these alleles were very low in four loci (WFT20: 0.088; WFT66: 0.088; WFT87: 0.144 and WFT139: 0.021). Besides the three mutation patterns mentioned above, base substitutions in the repeat or the flanking region were also observed in 9 and 9 loci respectively. They did not contribute to the length changes of the microsatellites. In addition, several loci had multiple mutation types mentioned above, e.g., WFT37 contained both base substitutions in the flanking region and step-wise mutation in the repeat region; WFT104 had both indels in the flanking regions and step-wise mutation in the repeat motif. A minimum number of contiguous repeats might be necessary for slippage to occur. These have been suggested to be four in di-nucleotide repeats and two in tri- and tetra-nucleotide repeats [21,22]. Using these criteria, we calculated the frequency of slippage consistent and inconsistent mutations. The allele size variation mainly came from the slippage at the repeat motifs. Sixty-six alleles with a frequency of 75.5% from the 18 loci possessed this mutation mechanism. Twenty-two alleles from 7 loci showed slippage in the flanking region, with the frequency of 19.9%. In addition, mutation mechanisms other than slippage also occurred in our microsatellites, with the frequencies of 12.6% (20 alleles from 6 loci) in the repeat motifs and 8.4% (11 alleles from 7 loci) in the flanking region. Generally speaking, slippage in the repeat motif and flanking region was the main mutation mechanism for the newly developed microsatellites.

Length changes in microsatellite DNA are generally thought to arise from replication slippage [9]. However, a complex mutational pattern of *F. occidentalis* EST-SSRs was observed in this study. These mutational patterns (changes in the number of microsatellite repeat units, base substitutions and indels within flanking region) were also found in microsatellites of insects [15,23] and other organisms, including the maize [24] and birds [25]. It seems that the complex mutational pattern is common in the eukaryotic genomes. Zhu *et al*. showed that indel slippage or length independent slippage tended to duplicate short sequences [26]. The number of repeat motifs of *F. occidentalis* ESTs was low (*n* < 9; Table 1) suggesting that indel slippage may be responsible for the complex mutational pattern of EST-SSRs in *F. occidentalis*.

Global and pairwise *F*_ST_ and *R*_ST_ among three populations were then calculated. *F*_ST_ assumes an infinite allele model and *R*_ST_ assumes a stepwise mutation model [27]. Global *F*_ST_ and *R*_ST_ considering all 18 loci showed a low but significant differentiation (global *F*_ST_ = 0.029, *P* < 0.001; global *R*_ST_ = 0.023, *P* < 0.001) among all three populations (Table 5). Moreover, including the loci which have one SSR or (and) indels in the flanking region did not significantly change the global *F*_ST_ and *R*_ST_ values with overlapping 95% confidence intervals (Table 5). When considering the same loci combinations, the global and pairwise *F*_ST_ and *R*_ST_ values did not differ significantly from each other with overlapping 95% confidence intervals (Table 5). However, no clear correlation was found between pairwise estimate of *F*_ST_ and *R*_ST_ (Spearman *r* = −0.202; *P* = 0.264). Pairwise *F*_ST_ results are quite consistent in all cases, Dali/Guiyang exhibited the lowest differentiation estimates and Harbin/Guiyang exhibited the highest. However, they were not reflected in the pairwise *R*_ST_ results. This might be due to the fact that the microsatellites mutated in the flanking region did not strictly conform to IAM and/or SMM model(s). Eleven out of 18 loci showed multiple sources of length variation which cannot be explained solely by gain or loss of one or two repeats as in the case of SMM based models. Thus, methods based on the IAM might be appropriate for many loci in our study, although they were not supported by our analysis. Anderson also suggested that IAM was more appropriate for one parasite’s (*Plasmodium falciparum*) microsatellites which have complex mutation patterns [28]. Furthermore, the precision of global differentiation estimates improves (the confidence intervals narrows) with increasing numbers of loci analyzed (Table 5). Thus, if users of the described microsatellites want precision in their estimates, more loci should be used.

## 3. Experimental Section

### 3.1. EST Database Mining

13,839 *F. occidentalis* EST sequences were obtained from GenBank [29]. EST-trimmer [30] was then used to remove poly (A/T) stretches from the 5′or 3′ ends until there were no (A)5 or (T)5 within the range of 50 bp. EST sequences shorter than 100 bp were excluded and those longer than 700 bp were clipped at their 5′ end to preclude the inclusion of low-quality sequences [31]. Those obtained sequences were screened for microsatellites containing at least five di-, five tri-, four tetra-, four pentaand four hexa-nucleotide repeats using the software MISA [14]. PCR primers flanking the microsatellite repeats were designed using Primer Premier 5.0 [32]. The selected ESTs were compared to the NCBI nr protein database using the BLASTX program. A suggested cut-off value of 1e-5 was chosen to assign a potential homologue for each EST sequence [13].

### 3.2. Sample Collection and DNA Extraction

In total, 96 *F. occidentalis* female adults were sampled representative of 3 sites in China during July 2010 to July 2011 (Table 3). Total genomic DNA was extracted by homogenizing a single female adult in a 50 μL mixture of STE buffer (100 mM NaCl, 10 mM Tris-HCl, 1 mM EDTA, pH 8.0) in a 1.5 mL Eppendorf tube. The mixture was incubated with 2 μL proteinase K (10 mg/mL) at 37 °C for 30 min, followed by 5 min at 95 °C. The samples were centrifuged briefly, and used immediately or stored at −20 °C for the PCR reactions.

### 3.3. Primer Testing

The forward primer of each set was tailed with U19 (GGTTTTCCCAGTCACGACG) to facilitate labeling. PCR amplifications were performed on an Applied Biosystems VeritiTM Thermal Cycler (Applied Biosystems). Each 10 μL amplification mixture contained 1 × PCR buffer, 0.2 mM of each dNTP, ~50 ng of DNA, 0.25 units of Maxima Hot Start Taq DNA polymerase (Fermentas, Canada), 0.04 μM of each forward primer, 0.2 μM of each of the reverse primer and the dye-labeled U19 primer (FAM, VIC, NED or PET). These cycling conditions were an initial denaturing for 4 min at 95 °C; 10 cycles of 95 °C for 30 s, 51 °C for 30 s, 72 °C for 30 s; 25 cycles of 95 °C for 30 s, 54 °C for 30 s, 72 °C for 30 s, and a final extension at 72 °C for 10 min. PCR products were run on the ABI 3130 capillary sequencer along with the GeneScan-500 LIZ size standard and allele sizes were determined using GENEMAPPER version 4.0 (Applied Biosystems).

### 3.4. Allele Sequencing

Different alleles per locus detected by the capillary sequencer were amplified using a 50 μL PCR reaction with non-fluorescent labeling primers (conditions as above with a specific anneal temperature at 52 °C). The purified PCR products (purified using Axygen cleanup kit) were subsequently ligated into the pGEM-T vector (Promega) and introduced into *Escherichia coli* DH5α cells. Six positive clones for each allele were sequenced to exclude PCR artefacts. Alignments of the sequenced alleles were generated using the Clustal X 2.0.11 program [33]. Several loci were then manually aligned using BioEdit 7.0.4 [34].

### 3.5. Data Analysis

All genetic statistics were carried out based on the genotyping data from three populations. MICRO-CHECKER 2.2.3 was used to detect genotyping errors due to null alleles, stuttering, or allele dropout using 1000 randomizations [20]. The program Genepop 4.0.10 [35] was used to test for linkage disequilibrium between pairs of loci in each population (100 batches, 1000 iterations per batch) and for deviations from Hardy–Weinberg equilibrium (HWE) at each locus/population combination using Fisher’s exact tests. The population genetic diversity indices such as total alleles per locus (*N*_A_), observed heterozygosity (*H*_O_), expected heterozygosity (*H*_E_) and mean number of alleles (*N*a) was assessed using GenAlEx 6.41 [36]. We also calculated the polymorphism information content (PIC) using CERVUS version 3.0 [37]. Pairwise *F*_ST_ and *R*_ST_ value and their significance for each population comparison were calculated with 10,000 permutations in Arlequin 3.0 [38] and RST CALC 2.2 [27], respectively.

## 4. Conclusions

In summary, 18 highly polymorphic EST-SSRs have been specifically developed for *F. occidentalis* in this study. Sequence analysis of alleles per locus revealed a complex mutational pattern of these EST-SSRs. Thus, these EST-SSRs are useful markers for the invasive species *F. occidentalis* but greater attention should be paid to the mutational characteristics of these markers when they are used in population genetic studies.

## Supplementary Materials



## Figures and Tables

**Table 1:** Frequency and distribution of SSRs in the analyzed *Frankliniella occidentalis* ESTs.

Repeats	Number of repeat motif (*n*)					

	4	5	6	7	8	Total
AC/GT	-	89	14	4	3	110
AG/CT	-	67	11	6		84
AT/AT	-	54	4	1		59
CG/CG	-	12				12
AAC/GTT	-	7	1			8
AAG/CTT	-	1				1
AAT/ATT	-	5	1			6
ACC/GGT	-	4	1			5
ACT/AGT	-	1				1
AGC/CTG	-	5				5
AGG/CCT	-	1	1			2
ATC/ATG	-	1				1
AAAC/GTTT	3					3
AAAT/ATTT	3					3
AACC/GGTT	2					2
AATC/ATTG	2					2
AATG/ATTC	3					3
ACAT/ATGT	1					1
ACTG/AGTC	1					1
AGAT/ATCT	3					3
ATGC/ATGC	1					1
ACGGC/CCGTG	1					1
NN(DNR)	-	222	29	11	3	265
NNN(TNR)	-	25	4			29
NNNN(TTNR)	19					19
NNNNN(PNR)	1					1

NN(DNR) is dinucleotidic repeats; NNN(TNR) is trinucleotidic repeats; NNNN(TTNR) is tetranucleotidic repeats; NNNNN(PNR) is pentanucleotidic repeats.

**Table 2:** Characteristics of 18 new EST-SSRs in *Frankliniella occidentalis*.

Locus	Genbank number (Sequence length)	Putative gene function a	Repeat motif	Primer sequence (5′-3′)	Size range (bp)	*T*a (°C)	*H*_O_	*H*_E_	*N*	*N*a	PIC
**WFT20**	GT306150 (292bp)	XP_001945214.1|PREDICTED: uncharacterized protein C14orf138-like [*Acyrthosiphon pisum*] 4e-08	(AC)6	F: CGTAGCCGCCCAAACTGTT R: CCTTCCAATTCAAATTCCCT	144–153	52	0.654	0.657	96	5	0.609
**WFT24**	GT303793 (745bp)	Unknown	(TG)6	F: ACGAAGTTTGGTTTGGGTGG R: AAGTTTCCTCCGCTCATTTC	208–214	52	0.257	0.290	96	2	0.258
**WFT25**	GT303588 (608bp)	Unknown	(GA)7	F: CACCAGTCGCGTTCATTGA R: GCCTCCAGCAGCACAAGTA	96–149	52	0.707	0.851	96	14	0.860
**WFT28**	GT303349 (784bp)	Unknown	(TA)6	F: GGGCTTGAAATAATGTTCTG R: GTAAATAAATCAGTGGAGGGT	91–95	52	0.241	0.224	96	3	0.230
**WFT37**	GT302424 (759 bp)	Unknown	(TTA)5	F: GCATACCCTGTGAACGAGTG R: ACAGAAGCAAATGTCTACCTGA	213–222	52	0.372	0.410	94	4	0.384
**WFT50**	GT310239 (785 bp)	Unknown	(TG)7	F: CGGAGTGAGCAGGAGTTGT R: TTGCCCCTACCAAAATATGA	171–181	52	0.313	0.458	95	6	0.427
**WFT51**	GT310133 (741bp)	XP_002425957.1| abrupt protein, putative [*Pediculus humanus corporis*] 7e-76	(TG)8	F: GTACGCAGGAGAAGTAAATG R: ACAAATCCAGATGGCAACC	297–305	52	0.623	0.590	96	5	0.555
**WFT64**	GT311293 (518 bp)	Unknown	(TCC)6	F: CTTTTCGGATTCTCCTTCG R: GGAGACCTGATTCACCGTATG	243–250	52	0.590	0.539	95	4	0.443
**WFT66**	GT305093 (175 bp)	Unknown	(ACT)5	F: AACTTAGGAAGAAAGACTGTAGA R: TGTTTACGCACGCACGCAT	113–116	52	0.505	0.476	96	3	0.432
**WFT83**	GT304065 (616 bp)	Unknown	(TA)5	F: GGAGGTACTGACTAAAGCATG R: GGGACAGACAAAACAGGAAA	172–176	52	0.462	0.451	96	4	0.394
**WFT87**	GT303951 (830 bp)	Unknown	(TG)5	F: GGTCTGAACTGTATGGGATG R: CAGGACCCTAGTATGTAAGAAA	259–277	52	0.346	0.391	94	7	0.394
**WFT98**	GT299180 (436 bp)	Unknown	(TG)5	F: GGGGCAGTTTGCTCTTGT R: CTGTTCATGGTCACTTTGG	145–153	52	0.645	0.636	96	5	0.587
**WFT104**	GT298583 (602 bp)	XP_002063687.1| GK15779 [*Drosophila willistoni*] 4e-17	(GT)5	F: TCACGCAAGCTAACAGCCCT R: ACAAAGTTGCCTGCCTGAAT	150–159	52	0.478	0.473	96	4	0.427
**WFT108**	GT300460 (686 bp)	Unknown	(AT)5	F: AGGATAGCTTGTTTTGTTGG R: CCATTTGTAACTAGCGTAGGA	135–140	52	0.355	0.350	96	4	0.328
**WFT124**	GT311015 (1114 bp)	Unknown	(TG)5	F: CATTATGTGCCTCACCTCCG R: GCCTCAATTCTTCCTTGCG	278–281	52	0.521	0.664	95	4	0.626
**WFT139**	GT301579 (439 bp)	Unknown	(TC)5	F:CATGGGTCCTTCCAGTGAG R: GCGAAACCTATCCCCTTATC	138–142	52	0.072	0.089	96	3	0.088
**WFT141**	GT310355 (612 bp)	Unknown	(GT)5	F:GCTTTTGCATACCTTGTCTTC R: GGTAAGGGCCGGTTTTGTT	174–183	52	0.588	0.678	96	7	0.651
**WFT144**	GT310315 (766 bp)	Unknown	(GT)5	F:TCGCAGAAGTTTGTGGTGAG R: GAGCCGATAAAAGTAGTGGAG	94–137	52	0.598	0.844	94	15	0.875
**Mean**							0.463	0.504		5.500	0.476

Ta, annealing temperature; *N*: number of analyzed individuals; *N*a: number of alleles detected; *H***_O_**: observed heterozygosity; *H***_E_**: expected heterozygosity; PIC: polymorphism information content;

a Based on BLASTX analysis. The species source and Accession No. of the best hit(s) is indicated, together with the E-value for the match.

**Table 3:** Collection information for samples used in this study.

Location	Nb samples	Coordinates	Sampling dates	Host
Harbin	31	45°44′51.13″N, 126°38′2.54″E	23 July 2010	*Tagetes erecta* L.; *Hosta ventricosa* (Salisb.) Stearn
Dali	35	25°36′17.49″N, 100°14′49.75″E	30 July 2011	*Petunia hybrida* Vilm; *Nicandra physalodes; Canna indica* L.
Guiyang	30	26°37′40.97″N, 106°49′23.06″E	26 July 2011	*Solanum melongena* L.; *Cucurbita pepo* L.

Nb samples, number of samples.

**Table 4:** Population genetic parameters at 18 microsatellite loci in 3 *Frankliniella occidentalis* populations.

Locus	Harbin					Dali					Guiyang				
	
	*N*	*N*a	*H*_O_	*H*_E_	*r*	*N*	*N*a	*H*_O_	*H*_E_	*r*	*N*	*N*a	*H*_O_	*H*_E_	*r*
**WFT20**	31	4	0.613	0.618	−0.003	35	5	0.714	0.671	−0.039	30	5	0.633	0.684	0.035
**WFT24**	31	2	0.452	0.437	−0.017	35	2	0.086	0.133	0.110	30	2	0.233	0.299	0.095
**WFT25**	31	10	0.613	0.811	0.119	35	11	0.743	0.871	0.069	30	12	0.767	0.873	0.064
**WFT28**	31	3	0.290	0.252	−0.155	35	3	0.400	0.355	−0.062	30	3	0.033	0.065	0.149
**WFT37**	30	3	0.200	0.238	0.062	35	3	0.400	0.483	0.078	29	4	0.517	0.510	0.007
**WFT50**	31	6	0.290	0.409	0.112	34	5	0.382	0.476	0.076	30	5	0.267	0.491	**0.193**
**WFT51**	31	4	0.613	0.558	−0.069	35	5	0.657	0.616	−0.040	30	5	0.600	0.596	−0.012
**WFT64**	31	3	0.548	0.529	−0.019	34	3	0.588	0.543	−0.056	30	3	0.633	0.546	−0.084
**WFT66**	31	3	0.387	0.395	0.013	35	3	0.629	0.568	−0.049	30	3	0.500	0.466	−0.023
**WFT83**	31	3	0.548	0.505	−0.047	35	4	0.371	0.348	−0.049	30	3	0.467	0.499	0.036
**WFT87**	30	5	0.200	0.187	−0.102	34	6	0.471	0.501	0.009	30	6	0.367	0.484	0.106
**WFT98**	31	4	0.516	0.546	0.027	35	4	0.686	0.644	−0.043	30	5	0.733	0.718	−0.011
**WFT104**	31	3	0.419	0.370	−0.064	35	4	0.514	0.573	0.051	30	4	0.500	0.474	−0.027
**WFT108**	31	3	0.355	0.297	−0.193	35	4	0.343	0.394	0.093	30	3	0.367	0.359	0.026
**WFT124**	31	4	0.452	0.618	0.129	34	4	0.412	0.654	0.176	30	4	0.700	0.722	0.019
**WFT139**	31	3	0.097	0.151	0.114	35	3	0.086	0.083	−0.043	30	2	0.033	0.033	−0.017
**WFT141**	31	5	0.516	0.668	0.103	35	7	0.514	0.601	0.069	30	5	0.733	0.766	0.020
**WFT144**	31	11	0.645	0.838	0.116	34	13	0.529	0.881	**0.196**	29	11	0.621	0.812	0.124
**Mean**		4.389	0.431	0.468	0.007		4.944	0.474	0.522	0.030		4.722	0.484	0.522	0.039

*N*: number of analyzed individuals; *N*a: number of alleles detected; *H***_O_**: observed heterozygosity; *H***_E_**: expected heterozygosity; *r*: null allele frequency; Bold indicates deviations from Hardy–Weinberg equilibrium after sequential Bonferroni correction for multiple tests (*P* < 0.05).

**Table 5:** Pairwise *F*_ST_ (below the diagonal) and *R*_ST_ (above the diagonal) matrix using different combinations of EST-SSRs of *Frankliniella occidentalis*.

Number of loci	Populations	Populations			Global *F*_ST_	Global *R*_ST_

		Harbin	Dali	Guiyang		
7 Loci a	Harbin		**0.025** (0.008–0.074)	0.006 (−0.006–0.066)	**0.022** (0.007–0.044)	**0.020** (0.011–0.067)
	Dali	**0.033** (0.007–0.074)		**0.021** (0.007–0.071)		
	Guiyang	**0.034** (0.016–0.049)	0.005 (−0.010–0.026)			
11 Loci b	Harbin		**0.024** (0.013–0.068)	0.020 (0.010–0.069)	**0.030** (0.011–0.056)	**0.030** (0.023–0.069)
	Dali	**0.031** (0.008–0.057)		**0.040** (0.026–0.087)		
	Guiyang	**0.032** (0.021–0.041)	**0.029** (0.002–0.085)			
14 Loci c	Harbin		**0.023** (0.015–0.061)	0.008 (0.002–0.051)	**0.025** (0.015–0.036)	**0.016** (0.014–0.051)
	Dali	**0.031** (0.014–0.055)		0.011 (0.007–0.052)		
	Guiyang	**0.033** (0.019–0.048)	**0.012** (−0.001–0.027)			
18 Loci	Harbin		**0.022** (0.017–0.056)	**0.016** (0.011–0.056)	**0.029** (0.016–0.046)	**0.023** (0.022–0.057)
	Dali	**0.030** (0.014–0.047)		**0.025** (0.019–0.063)		
	Guiyang	**0.032** (0.020–0.045)	**0.026** (0.004–0.062)			

a including the 7 loci mutated in the repeat motif;

b including the 7 loci mutated in the repeat motif and 4 loci which have one SSR in the flanking region;

c including the 7 loci mutated in the repeat motif and 7 loci which have indels in the flanking region; Bold indicates significant after Bonferroni correction (*P* = 0.05); Values in parentheses indicate 95% confidence intervals.
