# Supplementary material for: Development and Characterization of 18 Novel EST-SSRs from the Western Flower Thrips, Frankliniella occidentalis (Pergande)
Source: Int J Mol Sci. 2012 Mar 5;13(3):2863–76. doi: 10.3390/ijms13032863 (PMC3317692; doi:10.3390/ijms13032863)
Supplement: Supplementary file 1 [file ijms-13-02863-s001.pdf]

Supplementary Materials

Figure S1. Five remaining EST-SSRs mutated in the repeat motif in *Frankliniella occidentalis*.

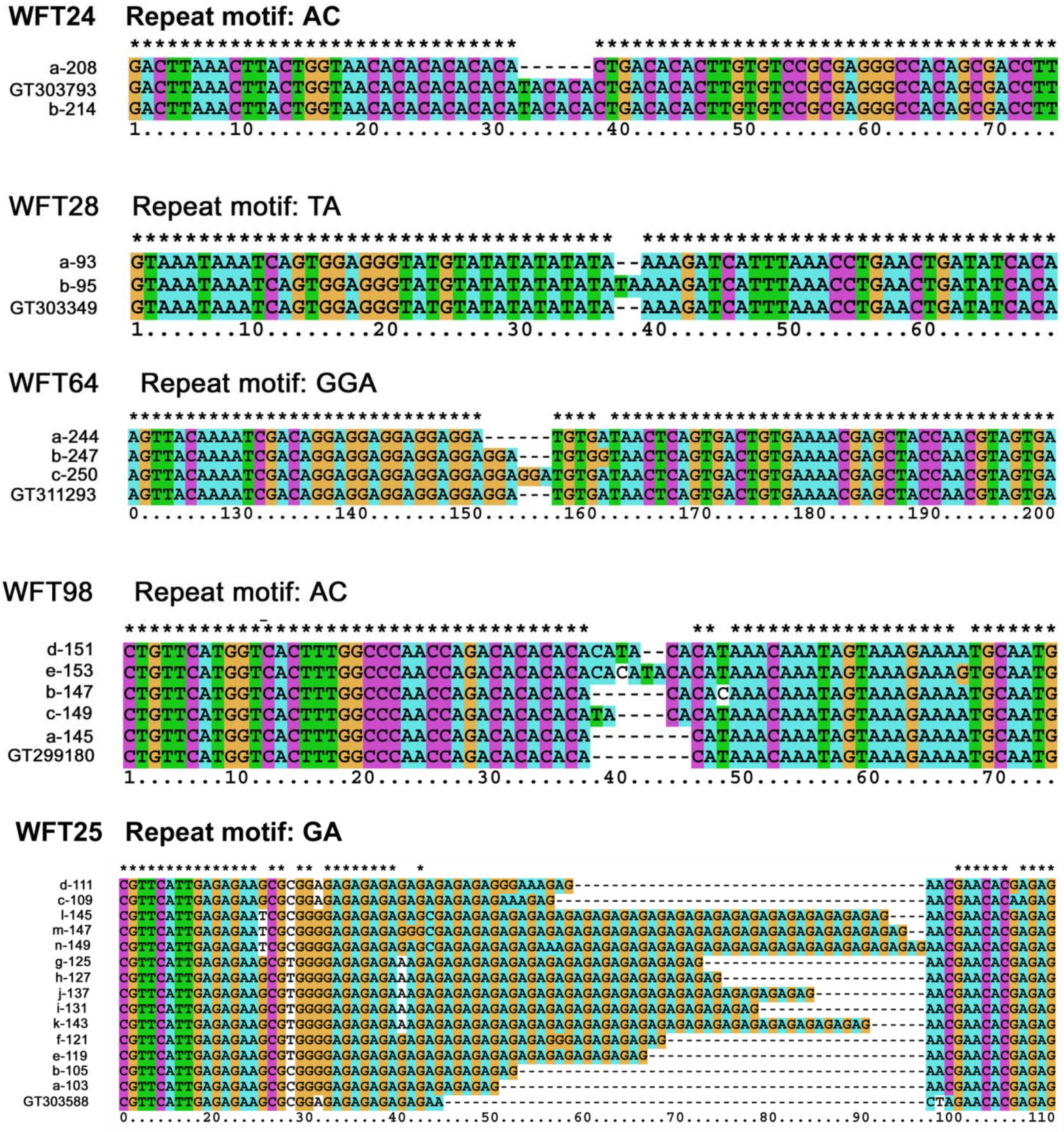

Figure S2. Mutational patterns of EST-SSRs in *Frankliniella occidentalis*. (**A**) microsatellites which have one SSR in the flanking region; (**B**) microsatellites which have indels in the flanking region.

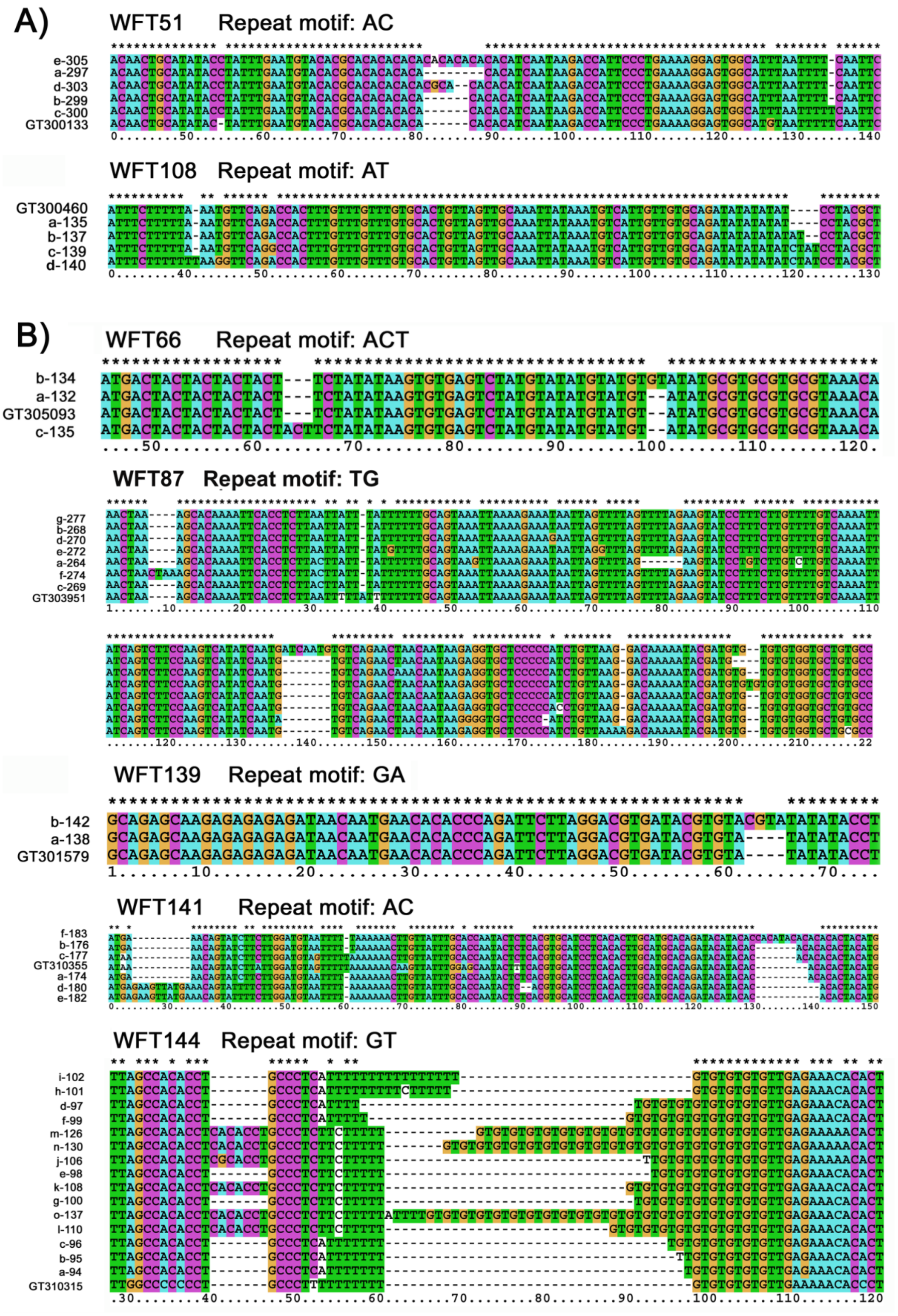

© 2012 by the authors; licensee MDPI, Basel, Switzerland. This article is an open access article distributed under the terms and conditions of the Creative Commons Attribution license (http://creativecommons.org/licenses/by/3.0/).
